# Effects of sponge‐to‐sponge contact on the microbiomes of three spatially competing Caribbean coral reef species

**DOI:** 10.1002/mbo3.1354

**Published:** 2023-04-27

**Authors:** Shelby E. Gantt, Patrick M. Erwin

**Affiliations:** ^1^ Center for Marine Science and Department of Biology and Marine Biology University of North Carolina Wilmington Wilmington North Carolina USA; ^2^ Present address: Department of Biology University of Alabama at Birmingham Birmingham Alabama USA

**Keywords:** allelopathy, chemical defense, competition, HMA‐LMA, morphology

## Abstract

Sponges perform important ecosystem functions, host diverse microbial symbiont communities (microbiomes), and have been increasing in density on Caribbean coral reefs over the last decade. Sponges compete for space in coral reef communities through both morphological and allelopathic strategies, but no studies of microbiome impacts during these interactions have been conducted. Microbiome alterations mediate spatial competition in other coral reef invertebrates and may similarly impact competitive outcomes for sponges. In this study, we characterized the microbiomes of three common Caribbean sponges (*Agelas tubulata*, *Iotrochota birotulata*, and *Xestospongia muta*) observed to naturally interact spatially in Key Largo, Florida (USA). For each species, replicate samples were collected from sponges in contact with neighbors at the site of contact (contact) and distant from the site of contact (no contact), and from sponges spatially isolated from neighbors (control). Next‐generation amplicon sequencing (V4 region of 16S rRNA) revealed significant differences in microbial community structure and diversity among sponge species, but no significant effects were observed within sponge species across all contact states and competitor pairings, indicating no large community shifts in response to direct contact. At a finer scale, particular symbiont taxa (operational taxonomic units at 97% sequence identity, OTUs) were shown to decrease significantly in some interaction pairings, suggesting localized effects for specific sponge competitors. Overall, these results revealed that direct contact during spatial competition does not significantly alter microbial community composition or structure of interacting sponges, suggesting that allelopathic interactions and competitive outcomes are not mediated by microbiome damage or destabilization.

## Graphical abstract

We conducted an investigation of microbiome impacts during spatial competition among coral reef sponges. Microbiome diversity and composition in zones of direct contact were indistinguishable from no‐contact zones and control sponges. These findings indicate that allelopathic interactions and competitive outcomes in sponges are not mediated by microbiome damage or destabilization.

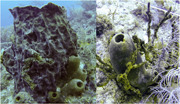

## 1 INTRODUCTION

Sponges represent a diverse invertebrate lineage containing over 8,500 identified species (Van Soest et al., 2012). They are sessile organisms recognized for their remarkable filtering processes (Milanese et al., 2003) and are known to possess symbiotic microorganisms capable of nutrient cycling within their mesohyl (Bayer et al., 2014; Hoffmann et al., 2009; Schläppy et al., 2010). Accordingly, the sponge and its complex microbial community (“microbiome”) have been used to address ecological questions pertaining to the sponge host and its environmental impacts. Furthermore, sponges have shown trends of increasing in biomass on Caribbean coral reefs over the last decade (McMurray et al., 2010), are already dominant community members (60% of reef cover) on some Caribbean reefs (Loh et al., 2015), and are known to compete allelopathically with other coral reef species (Chadwick & Morrow, 2011; Slattery & Lesser, 2021). Thus, understanding how sponges interact with other coral reef community members, including other sponge species, is important for future conservation efforts and predictive forecasts in coral reef communities.

Previous work on Caribbean coral reefs has shown that most sponges grow in contact with (28.6%) or in proximity to (31.0%) other sponges, with the remaining individuals (40.4%) observed growing in isolation (Engel & Pawlik, 2005). Such direct contact or proximity to neighboring sponge individuals can result in tissue damage, impeded growth, and over‐growth as the organisms compete for space and ambient resources. These interactions are mediated by differential sponge growth rates, chemical defenses (i.e., allelopathy), and ambient predation pressures. Indeed, predation levels can interact with spatial competition pressures, such as physical or chemical defenses utilized by the host to deter sponge predation (Chanas & Pawlik, 1996; Pawlik et al., 1995; Uriz et al., 1996) may also represent allelopathic chemicals that assist in spatial competition with other sponge species or corals (Engel & Pawlik, 2000; Pawlik et al., 2007; Porter & Targett, 1988). Previous work has suggested that sponges vary in allelopathic chemical defenses on a species‐by‐species basis (Assmann et al., 2004; Engel & Pawlik, 2000; Proksch, 1994; Waddell & Pawlik, 2000) and that intraspecific variation in defenses occurs within some sponge species (Chanas & Pawlik, 1997). These intraspecific variations in allelopathy do not correlate with the sponge's size or its ability to compete spatially (Chanas & Pawlik, 1997) but may be affected instead by the level of predation within the sponge's environment.

Sponge microbiome characterization is increasingly being incorporated into the study of sponge health and ecological function, facilitated by more affordable and rapid DNA sequencing technologies. Previous research has shown that sponges host abundant and complex microbial communities (Taylor et al., 2007, 2013; Thomas et al., 2010, 2016) that are distinct from the free‐living microbial assemblages (Gantt et al., 2017; Hentschel et al., 2002; Weigel & Erwin, 2016). These microbial communities are generally sponge species‐specific, even across great distances (Hentschel et al., 2002; Lee et al., 2011), and host sponges exist in two main categories based on the abundance and diversity of their associated microbes (Gloeckner et al., 2014; Poppell et al., 2014; Schöttner et al., 2013): high microbial abundance (HMA) sponges that contain 10^8^–10^10^ bacteria cells per gram of sponge (2–4 orders of magnitude greater than seawater, Hentschel et al., 2006) and low microbial abundance (LMA) sponges that host microbial communities at concentrations similar to seawater (10^6^–10^8^ bacteria cells per gram of sponge, Hentschel et al., 2006). Previous studies have applied microbial community analyses to assess sponge health (Webster et al., 2002), the role of sponges in nutrient cycling within coral reef communities (Gantt et al., 2019; Hoffmann et al., 2009; Rix et al., 2016), and climate change impacts on sponge functioning and survival (Lemoine et al., 2007; Lesser et al., 2016). Despite the importance of the sponge microbiome to host health and ecology (Pita et al., 2018; Slaby et al., 2019) and contributions to secondary metabolite synthesis (Helber et al., 2019; Liu et al., 2019), the impact of spatial competition on the structure of microbial communities in sponges has not been investigated.

To characterize the ecological aspects of sponge communities more fully in coral reef ecosystems, the current study investigated microbial community effects from the spatial competition (i.e., direct contact) among interacting sponge species. In this study, we posed three hypotheses: (1) sponge microbiomes will differ among host species, (2) sponge microbiomes will differ within each host species between tissue in contact with neighboring sponges versus no contact and control tissues, and (3) intraspecific microbiome shifts will vary by host and competitor pairing. To test these hypotheses, sponge tissue was sampled from three common Caribbean sponges (*Agelas tubulata*, *Iotrochota birotulata*, and *Xestospongia muta*) observed to naturally interact spatially and microbial communities were characterized using partial 16S rRNA gene sequences (V4 region). Differences in sponge microbiomes were assessed at the community and operational taxonomic unit (97% sequence identity, OTU) levels for each interaction type and sponge competitor to assess the effects of sponge‐to‐sponge contact on the composition and structure of host‐associated microbial communities.

## 2 MATERIALS AND METHODS

### 2.1 Sample collection

**Figure 1:**
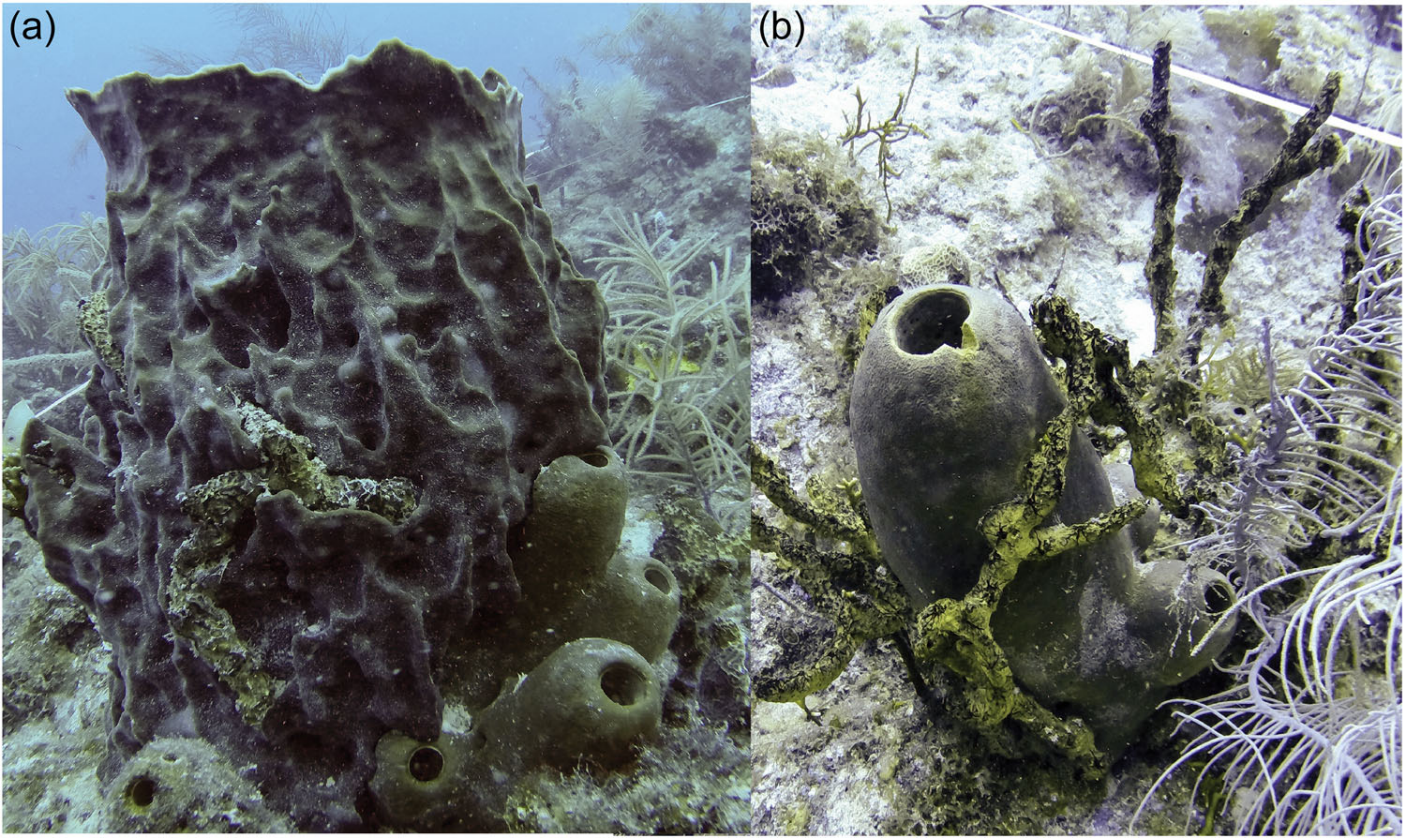
Photographs of spatial interactions among coral reef sponges. (a) *Xestospongia muta* (center, barrel sponge) overgrowing *Agelas tubulata* (bottom right, tube sponge) and *Iotrochota birotulata* (left center, rope sponge). (b) *A. tubulata* (center, tube sponge) in contact with *I. birotulata* (rope sponge).

Sponge tissue samples were collected from three common sponge species, representing two HMA species (*A. tubulata* and *X. muta*) and one LMA species (*I. birotulata*), observed to naturally interact on the reef (i.e., occurred in direct contact with each other, Figure 1). Triplicate individuals were collected for two interaction pairs (*A. tubulata‐I. birotulata* and *X. muta‐I. birotulata*) and one replicate for the third (*A. tubulata‐X. muta*, Table 1). For each direct‐contact replicate, two tissue areas were sampled: a “contact” site (i.e., area of tissue in contact between two spatially competing sponges) and a “no contact” site (area of tissue distant from the site of contact between two spatially competing sponges). “Contact” site tissues were easily discernable even following detachment of the competing sponges, due to clear morphological changes where the sponges interacted (*A. tubulata* = faded pigmentation, *I. birotulata* = blackened pigmentation with flattened and lipped surfaces, *X. muta* = faded pigmentation and flattened morphology). In addition, ‘control’ tissue samples were collected from triplicate individuals for each species that occurred in isolation (i.e., sponges not in contact with any neighboring sponges). All sponge tissue samples were collected between 12 and 23 m depth at Conch Reef, Florida, USA (24° 56.9′N, 80° 27.2′W) in June 2017. All sponges appeared healthy at collection, with no noticeable signs of disease, and were collected in separate sterile Whirl‐pak® bags. Following transfer to the laboratory, samples were rinsed with 95% ethanol, preserved in 100% ethanol in 1.5 mL tubes, and stored at −20°C until processing. Triplicate seawater samples (1L) were collected each day of sponge sampling and were concentrated onto 0.2 µm filters, frozen using liquid nitrogen, and stored at −20°C until processing.

**Table 1:** Alpha diversity metrics of sponge and seawater microbiomes by sponge abundance category (high microbial abundance [HMA] vs. low microbial abundance [LMA]) and interaction type (contact, no contact, control).

Source	Category	Interaction	*n*	S	H′	D
*Agelas tubulata*	HMA	Overall	11	766 ± 20	3.70 ± 0.06	0.050 ± 0.004
		Control	3	755 ± 55	3.70 ± 0.04	0.049 ± 0.003
		Contact	4	805 ± 13	3.83 ± 0.08	0.041 ± 0.004
		No contact	4	736 ± 34	3.58 ± 0.13	0.061 ± 0.009
*Iotrochota birotulata*	LMA	Overall	15	916 ± 53	1.62 ± 0.12	0.554 ± 0.033
		Control	3	811 ± 65	1.29 ± 0.10	0.654 ± 0.025
		Contact	6	1042 ± 112	1.86 ± 0.25	0.494 ± 0.064
		No contact	6	843 ± 33	1.55 ± 0.14	0.565 ± 0.041
*Xestospongia muta*	HMA	Overall	11	821 ± 17	4.25 ± 0.14	0.038 ± 0.009
		Control	3	837 ± 25	4.37 ± 0.21	0.028 ± 0.006
		Contact	4	843 ± 19	4.27 ± 0.13	0.033 ± 0.007
		No contact	4	786 ± 36	4.14 ± 0.35	0.051 ± 0.024
Seawater	‐‐‐	‐‐‐	9	899 ± 13	3.46 ± 0.02	0.103 ± 0.002

*Note*: “Overall” considers samples of all interaction types for the specified sponge species. Values are means ± 1 standard error. No significant differences across interaction types within each sponge were detected for any diversity metric. S, operational taxonomic units (OTUs) richness; H′, Shannon‐Weaver; D, Simpson diversity index.

### 2.2 DNA extraction and sequence processing

DNA extracts were prepared from sponge tissue (dissected into 2 mm^2^ cubes, including mesohyl and surface tissue, *n* = 38) and seawater filtrate (half of the seawater filter, *n* = 9) using the DNeasy® Blood and Tissue kit (Qiagen) following manufacturer's protocols. Partial (V4) 16S rRNA gene sequences were amplified using the 515F forward and 806R reverse primers (Caporaso et al., 2011). Amplicons were sequenced on an Illumina MiSeq platform at Molecular Research LP (Shallowater, TX) and raw sequence reads were processed in mothur (Schloss et al., 2009) using a modified bioinformatics pipeline as described previously (Weigel & Erwin, 2016). Briefly, raw sequences were demultiplexed, quality‐filtered, aligned, classified, and clustered into operational taxonomic units (OTUs) with 97% sequence identity. All sample sequences were subsampled to the lowest read count (*n* = 48,355) and all subsequent analyses were based on the subsampled data set.

### 2.3 Data analysis

#### 2.3.1 Microbial community diversity

Diversity for all samples was assessed using mothur (version 1.39.5) to calculate OTU richness (S), Shannon‐weaver diversity index (H’), and Simpson index (D). Two‐way crossed analyses of variance (ANOVA) were run in JMP (version 13.0.0) to test for significant differences in diversity indices across the factors of species (*A. tubulata*, *I. birotulata*, *X. muta*), interaction type (contact, no contact, control) and the interaction term (species*interaction type). Tukey's honest significant difference (HSD) test was run to test for multiple post‐hoc comparisons of means.

#### 2.3.2 Microbial community structure

Bray‐Curtis similarity matrices were constructed within Primer‐e (version 7.0.13) using square root transformed abundance data, to give rare taxa more representation within subsequent comparisons, and were visualized in a cluster‐based dendrogram. Permutational multivariate analyses of variance (PERMANOVA, version 1.0.1) were performed to assess differences in microbial community structure across the factor species (*A. tubulata*, *I. birotulata*, *X. muta*), interaction type (contact, no contact, control), and an interaction term. Pairwise PERMANOVA comparisons were run for significant factors, with Monte Carlo asymptotic *p*‐values used to determine pairwise significance.

#### 2.3.3 OTU level analyses

Similarity percentages (SIMPER) analysis was used with OTU relative abundance matrices and a cutoff of 0.70 to assess the specific OTUs of the abundant community driving the dissimilarity among the species and interaction type factors. Significant differences in OTU relative abundance were assessed using Metastats (White et al., 2009) as implemented in mothur with 1000 permutations. OTUs of interest were further characterized using the nucleotide‐nucleotide Basic Local Alignment Search Tool (BLASTn) in NCBI to compare 16S rRNA gene sequences against the GenBank database.

## 3 RESULTS

### 3.1 Microbial community diversity and composition

**Figure 2:**
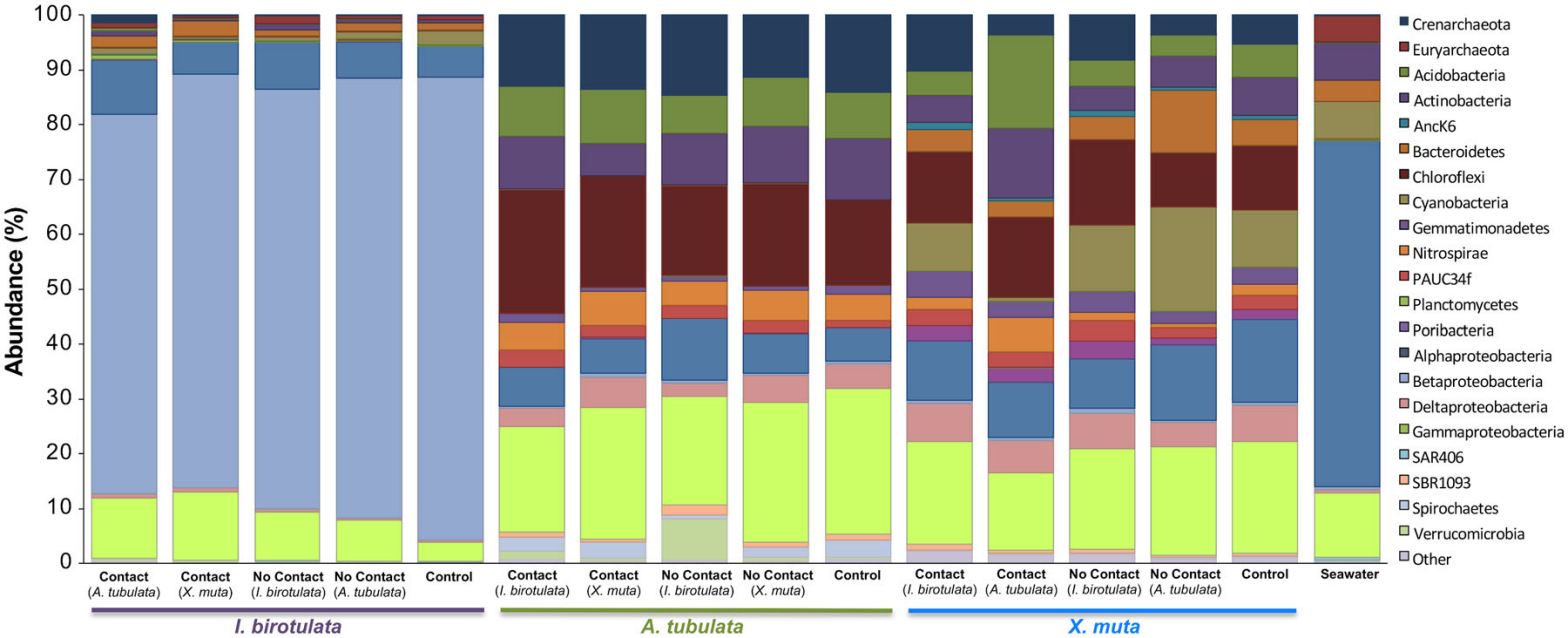
Phylum level composition of sponge and seawater microbiomes averaged by source and interaction type (*Agelas tubulata*, green; *Iotrochota birotulata*, purple; *Xestospongia muta*, blue). Class‐level taxonomy is shown for Proteobacteria to enhance the resolution of this dominant phylum.

From the 3.9 million sequences recovered, the bioinformatics analysis yielded 9206 OTUs representing 39 identified phyla, including three archaeal phyla. For all sponge species, the microbial communities in sponge contact pairs (considering tissue from contact and no contact sites) contained greater phylum‐level diversity (*A. tubulata* = 28, *I. birotulata* = 37, and *X. muta* = 27) than the control sponges (*A. tubulata* = 27, *I. birotulata* = 27, and *X. muta* = 24). Of the three archaeal phyla identified, Crenarchaeota and Euryarchaeota were found in the microbial communities of all sponge species and seawater, while Parvarchaeota were only consistently found within *X. muta* microbial communities, though in low relative abundance (0.008%–0.02% abundance across averaged interaction types and pairings). The microbiomes of the HMA sponges (*A. tubulata* and *X. muta*) showed a greater relative abundance of the phyla Crenarchaeota, Acidobacteria, Chloroflexi, and Nitrospirae than the LMA sponge (*I. birotulata*) microbial community (Figure 2). Additionally, a single betaproteobacterium (OTU001) comprised the majority of the *I. birotulata* microbial communities (65.8%–80.7% relative abundance, Table 2, Figure 2).

**Table 2:** Operational taxonomic units (OTUs)‐level effects of sponge‐to‐sponge contact on microbiomes within host species, showing OTUs that differed significantly in relative abundance (Metastats) across interaction types (contact, no contact, control) within each sponge species.

Host sponge (competitor)	OTU no.	BLASTn match (source, % identity)	SIMPER (%)	Metastats *p*‐value	Pairwise comparison (relative abundance)
*Agelas tubulata (Iotrochota birotulata)*	010	EF076162 Uncultured Gammaproteobacteria (*Agelas dilatata*, 100)	6.53	0.0053	Contact (5.6%) versus control (8.2%)
	014	EF076173 Uncultured Chloroflexi (*Agelas dilatata*, 99)	6.16	0.0239	Contact (5.9%) versus control (2.4%)
	062	EF076192 Uncultured Chloroflexi (*Agelas dilatata*, 99)	1.65	0.0208	Contact (1.4%) versus control (1.7%)
	062	EF076192 Uncultured Chloroflexi (*Agelas dilatata*, 99)	1.65	0.0277	Contact (1.4%) versus no contact (0.4%)
*I. birotulata (A. tubulata)*	001	EF657859 Uncultured Betaproteobacteria (*Eunicea fusca*, 97)	84.73	0.0225	Contact (65.8%) versus control (80.7%)
	011	EF657859 Uncultured Betaproteobacteria (*Eunicea fusca*, 97)	3.95	0.0315	Contact (3.0%) versus control (3.4%)
	011	EF657859 Uncultured Betaproteobacteria (*Eunicea fusca*, 97)	3.95	0.0339	Contact (3.0%) versus no contact (3.5%)
*I. birotulata (Xestospongia muta)*	002	MH077514 Uncultured Pelagibacteraceae (Seawater, 100)	2.50	0.0278	Contact (1.6%) versus no contact (3.5%)
*X. muta (I. birotulata)*	029	JN210798 Uncultured Xanthomonadales (*Rhopaloeides odorabile*, 99)	4.71	0.0500	Contact (1.9%) versus control (3.5%)
	094	JN596639 Uncultured Alphaproteobacteria (*Xestospongia muta*, 100)	1.38	0.0475	Contact (0.6%) versus control (1.1%)
	094	JN596639 Uncultured Alphaproteobacteria (*Xestospongia muta*, 100)	1.38	0.0435	Contact (0.6%) versus no contact (1.1%)
	097	JN596669 Uncultured Gammaproteobacteria (*Xestospongia testudinaria*, 97)	1.25	0.0240	Contact (0.8%) versus no contact (0.7%)

*Note*: The top BLASTn match, contribution to overall microbiome similarity within the host sponge species (SIMPER %), and average relative abundances across interaction types are given for each operational taxonomic units (OTUs). The *A. tubulata*–*X. muta* pairing did not have enough replicates for analysis.

Interspecific comparisons of the microbial community diversity indicated significant differences in OTU diversity (ANOVA, Shannon–Weaver *p* < 0.001, Simpson *p* < 0.001) and OTU richness (ANOVA, *p* = 0.014) among sponge species. No significant differences in intraspecific diversity among interaction types occurred for any diversity metric (Table 1).

### 3.2 Comparisons of microbial community structure

**Figure 3:**
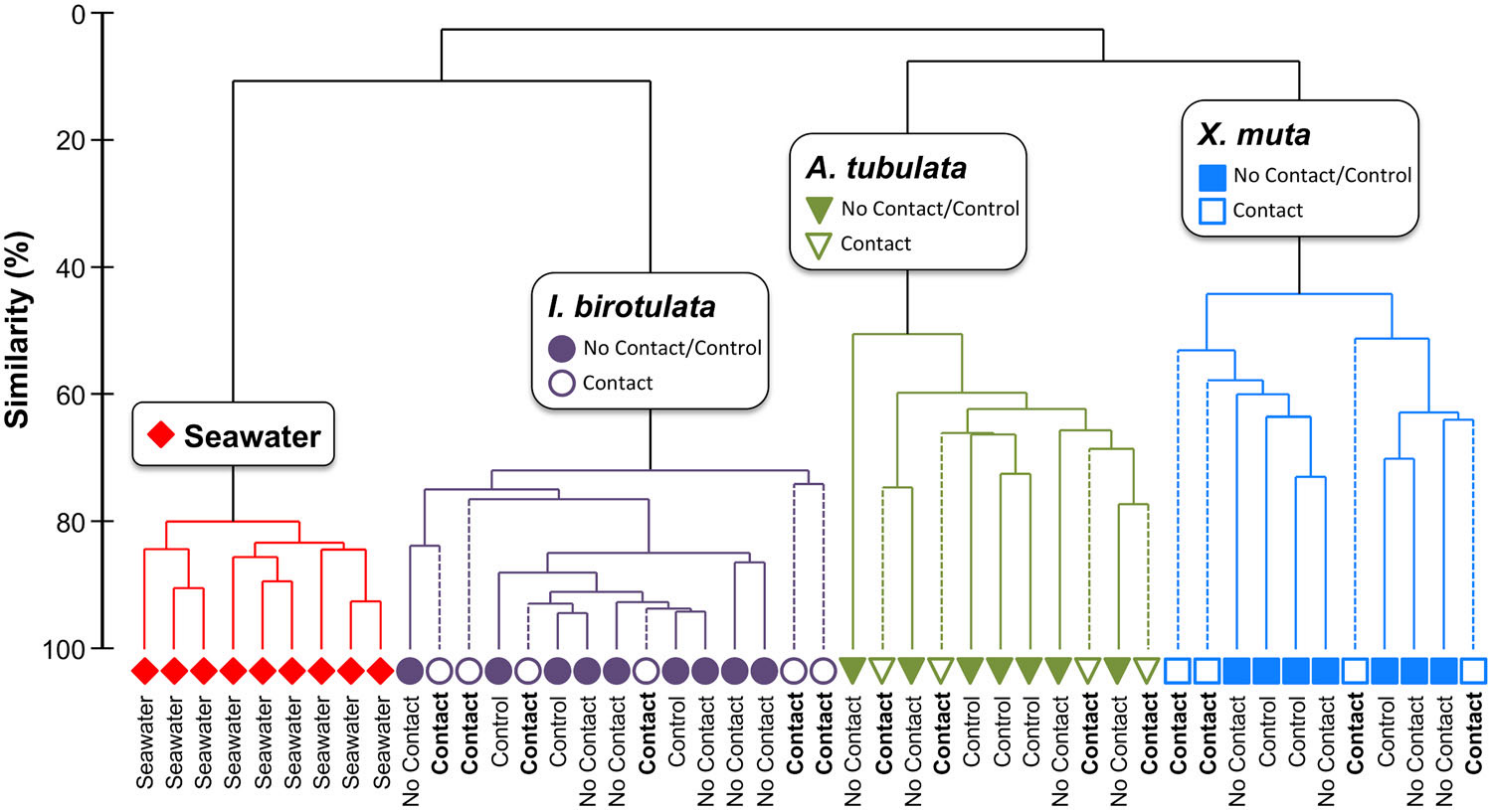
Cluster dendrogram of sponge and seawater microbiomes based on Bray‐Curtis similarity. Symbols denote sponge species (*Agelas tubulata*, green triangles; *Iotrochota birotulata*, purple circles; *Xestospongia muta*, blue squares) and seawater samples (red diamonds). Contact samples are highlighted by dashed lines, open symbols, and bold text. No significant differences in microbial community structure were detected across interaction types (contact, no contact, control).

Significant differences in microbial community structure were detected among sponge species (*p* = 0.001) but not across interaction types (contact, no contact, control, *p* = 0.340) or the interaction term (*p* = 0.241). Accordingly, host sponge species accounted for the majority of variation in sponge microbiomes (66.9%) and pairwise comparisons of all species pairs were significant (*p* = 0.001). In contrast, interaction type accounts for little microbiome variation among samples (2.2%). The similarity‐based dendrogram showed that samples clustered into two main branches: one consisting of the LMA sponge and seawater microbial communities, and the other consisting of the two HMA sponge microbial communities. Within each branch, the microbial communities are further clustered into species‐specific groups (Figure 3). Consistent with statistical analyses, no further clustering within species by interaction type was observed, except for control microbial communities in *A. tubulata* (Figure 3).

OTU‐level analyses identified nine microbial taxa that exhibited significant relative abundance shifts in sponge tissue contacting neighboring sponges, compared to no contact or control tissue of the same sponge species (Metastats, *p* < 0.05; Table 2). In most cases, these OTUs represented previously described low‐abundance members of the sponge microbiomes that decreased further in relative abundance in contact tissue samples. Consequently, the symbiont OTUs that contributed most to each sponge host's unique microbiome did not differ among interaction types. An exception was OTU 001 (Betaproteobacterium), the dominant symbiont in *I. birotulata* that significantly decreased from 80.7% relative abundance in control sponges to 65.8% in contact sponges (Table 2).

## 4 DISCUSSION

The microbial communities from each sponge species investigated (*A. tubulata*, *I. birotulata*, and *X. muta*) differed significantly from each other and from free‐living microbial communities in the surrounding environment, consistent with previous studies (Hentschel et al., 2012; Jackson et al., 2012). The LMA sponge, *I. birotulata*, hosted microbial communities more similar to seawater communities than those in HMA sponges (*A. tubulata* and *X. muta*), with significantly lower diversity and increased Proteobacteria presence than HMA counterparts, supporting results from past HMA‐LMA sponge studies and comparisons (Gantt et al., 2019; Giles et al., 2013; Gloeckner et al., 2014; Poppell et al., 2014). These characteristic patterns in sponge microbial communities are likely related to differences in host physiology and pumping rates between HMA and LMA sponges (Poppell et al., 2014; Weisz et al., 2008). Notably, distinct microbiomes were also observed between the two HMA hosts, indicating a strong influence of host species on microbial community structure.

Within each host sponge species, microbial community analyses revealed high microbiome stability across interaction types, suggesting that allelopathic interactions and spatial competition outcomes among coral reef sponges were not mediated by microbiome damage or destabilization. This is surprising since the microbial communities of some sponges are involved in allelopathic chemical production (Rust et al., 2020; Tianero et al., 2019) and microbiome disruption from spatial competition has been documented in corals (Pawlik et al., 2007; Thinesh et al., 2020; Vega Thurber et al., 2012). Of the sponges investigated herein, both *A. tubulata* and *X. muta* utilize allelopathy during the competition (Assmann et al., 2004; Kelly et al., 2003; Waddell & Pawlik, 2000), while *I. birotulata* is not known to utilize chemical defenses to deter predation or overgrowth (Engel & Pawlik, 2000; Pawlik et al., 1995). Our data show little effect of direct tissue contact on sponge microbial communities and suggest that direct damage to host cells is the primary mechanism of allelopathic interactions among sponges. In contrast, coral microbiome disruption appears to be an important mechanism of spatial interactions on reefs. For example, some macroalgae utilize dissolved organic carbon release to disrupt coral microbiomes (Smith et al., 2006; Vega Thurber et al., 2012) and harbor coral pathogens in their microbial communities that may aid in spatial competition (Barott et al., 2012). Direct contact with sponges can also disrupt coral microbiomes (Thinesh et al., 2020), further evidence linking microbiome stability and spatial interaction mechanisms on coral reefs.

These results follow a general pattern of greater microbiome stability in sponge hosts compared to coral counterparts. Coral microbiomes have been shown to change in response to temperature fluctuations (Maher et al., 2019), season (Glasl et al., 2020), pollution (Joyner et al., 2015), and disease (Slaby et al., 2019), among other factors. Sponge microbiomes (at least in shallow water habitats) have shown resistance to perturbations and stability in response to stressors such as pollution (Gantt et al., 2017), elevated temperatures (Luter et al., 2012; Pita et al., 2013), and ocean acidification (Kandler et al., 2018). Sponges also show strong stability in community composition and structure across seasons (Erwin et al., 2012, 2015), when losing or acquiring photosymbionts (Britstein et al., 2020) and during periods of food shortage stress (Pita et al., 2013). Results from the current study add allelopathic interactions to the growing list of environmental factors that do not alter sponge microbiomes and highlight differences in microbiome stability across coral reef species that may inform predictions of host success under changing environmental conditions.

While sponge microbiomes were stable at the community level, fine‐scale analyses revealed changes in the relative abundance of some symbiont OTUs across interaction types, primarily at contact sites. These trends indicate minor shifts in microbial communities when sponge tissue is in contact with other sponge competitors, as most OTUs that varied across interaction types were rare members of the sponge microbiomes. Only nine symbiont OTUs (of the >9,000 identified) varied significantly at contact sites and exhibited >1% relative abundance: two OTUs in the phylum Chloroflexi and seven OTUs in the phylum Proteobacteria. Some of these microbial shifts may result from altered physical (e.g., shading) and chemical conditions at contact sites. In *A. tubulata*, OTU010 (Gammaproteobacteria) decreased in relative abundance in contact tissue, while OTU014 (Chloroflexi) increased two‐fold in contact tissue. OTU010 was affiliated with Ectothiorhodospiraceae, a family of purple sulfur bacteria that utilizes photosynthesis (Henry & Cogdell, 2013), thus shifts in the abundance of this OTU may result from shading at contact sites. OTU014 was affiliated with SAR202, a group of heterotrophic, free‐living cells known to be sulfite‐oxidizers (Mehrshad et al., 2018). In *X. muta*, two proteobacterial OTUs (OTU029, 094) decreased in relative abundance in contact tissue, with OTU094 classified as Rhodospirillaceae, a family of purple non‐sulfur photosynthetic bacteria (Kim et al., 2011). Therefore, while the overall stability of microbial communities across interaction types supports minimal impacts of allelopathic competition on sponge microbiomes, particular OTUs may exhibit shifts from localized indirect impacts during direct tissue contact with competitors, such as shading and abrasion.

## 5 CONCLUSION

In summary, our data revealed that sponge‐to‐sponge spatial contact does not affect overall microbial community composition and structure for the investigated Caribbean host species, with only minor shifts (changes in individual OTUs) occurring from indirect impacts of the interaction (e.g., shading). These results highlight the stability of sponge microbial communities during spatial interactions and suggest that microbiome disruption is not the main mechanism of host damage from allelopathic interactions and has minimal impacts on spatial competition outcomes among Caribbean sponges. Future investigations targeting additional host sponge species and experimenting with forced interactions among sponges will provide additional insight into the interplay between microbiome structure, chemical defenses, and spatial competition in coral reef invertebrates. Ultimately, a clear understanding of sponge‐to‐sponge competition may yield insights into which species of sponges will dominate and shape future Caribbean reefs as corals continue to decline.

## AUTHOR CONTRIBUTIONS

**Shelby E. Gantt**: Conceptualization (lead); data curation (lead); formal analysis (lead); writing—original draft (lead); writing—review and editing (supporting). **Patrick M. Erwin**: Conceptualization (supporting); data curation (supporting); formal analysis (supporting); funding acquisition (lead); writing—original draft (supporting); writing—review and editing (lead).

## CONFLICT OF INTEREST STATEMENT

None declared.

## ETHICS STATEMENT

None required.

## Data Availability

The 16S rRNA sequence datasets generated and analyzed in the current paper are available in the NCBI Sequence Read Archives (Project: PRJNA462039): https://www.ncbi.nlm.nih.gov/bioproject/PRJNA462039
